# Psychometric evaluation of the Brazilian-Portuguese version of the Functional Outcome of Sleep Questionnaire 10 (FOSQ-10) in patients with obstructive apnea

**DOI:** 10.1016/j.bjorl.2024.101452

**Published:** 2024-06-04

**Authors:** Ana Paula Sereni Manfredi Moreira, Elisabete Raca Romero de Oliveira, Luciane Bizari Coin de Carvalho, Roberto Celso Colacioppo, Terri Weaver, Edilson Zancanella, Agrício Nubiato Crespo

**Affiliations:** a Universidade Estadual de Campinas (Unicamp), Campinas, São Paulo, Brazil; b Universidade Federal de São Paulo, São Paulo, SP, Brazil; c Anova Consulting (Brazil), Campinas, São Paulo, Brazil; d University of Illinois Chicago, Chicago, IL, US

**Keywords:** Functional outcomes of sleep questionnaire, FOSQ-10, Quality of life, Reliability and validity, Obstructive sleep apnea

## Abstract

**Objective:**

Perform the validation and psychometric evaluation of the Brazilian-Portuguese translation of the Functional Outcome of Sleep Questionnaire 10 (FOSQ10).

**Materials and methods:**

182 patients (65 females 48.3 ± 14.4 years and 117 males 46.9 ± 12.4 years), were evaluated by sleep physicians suspected of having Obstructive Sleep Apnea, underwent polysomnography and completed the FOSQ-10 and the Epworth Sleepiness Scale. APA & NCME, 2014 was used to validate the data as the American Educational Research Association recommended.

**Results:**

Quality indicators such as Bartlett's test of sphericity (χ^2^ = 1108.2; gL = 45; p =  0.000010) and KMO (0.83), and adherence measures, attest to the quality of the model. The indicators TLI (0.97), CFI (0.98), and RMSEA (0.04) fall within the expected values. Using the Eigenvalue > 1 technique, two factors explain 53% and 13.3% of the variances. In the Parallel Analysis technique, a single factor explained 59.4653% of the random variance, and the Unidimensionality indicators UniCo = 0.921, ECV = 0.822, and MIREAL = 0.253, were supported. Construct Validity: reliability coefficients Cronbach’s α = 0.87, McDonald’s ordinal Omega index 0.9, and the Composite Reliability 0.891 were satisfactory.

**Convergent validity:**

There was a significant Spearman correlation between FOSQ-10 and the Epworth Sleepiness Scale (r = 0.364 [−0.487; −0.226]).

**Criterion validity:**

Was not possible to differentiate the groups based on the severity of AHI using FOSQ-10P.

**Conclusions:**

The Brazilian translation of FOSQ-10 is valid and reliable for identifying significant effects of excessive daytime sleepiness in patients with Obstructive Sleep Apnea.

**Level of evidence:**

2.

## Highlights

• The translation and cultural adaptation of the FOSQ-10 into Portuguese is valid and reliable.
• The study methodology was grounded in the APA & NCME, 2014 guidelines proposed by the AERA.
• The missing values are non-random, associated with individuals not performing tasks (questions 3, 4, and 10).
• The total scores of FOSQ-10P exhibited a significant negative correlation with the total scores of the ESE.

## Introduction

Excessive Daytime Sleepiness (EDS) impairs daily functioning,^1^ reducing the ability to perform basic tasks, affecting cognitive efficiency, increasing the risk of accidents in various contexts, and significantly impacting public health.^2^ Additionally, drowsiness decreases engagement in social interactions and physical activities.

Health-Related Quality of Life (HRQoL)^3^ is a multidimensional concept that evaluates the long-term impact of diseases, disabilities, or disorders on an individual's life. It considers various sleep disturbances, such as excessive daytime sleepiness,^4^ as assessed by tools like the Epworth Sleepiness Scale (ESS)^5^ ‒ translated and validated for Brazilian Portuguese^6^ ‒ and Functional Outcome of Sleep Questionnaire (FOSQ-30).^7^ The FOSQ-10,^8^ a shorter version, is widely used in large-scale studies and clinical practice, measuring the impact of sleep-related dysfunction on daily activities efficiently. This study focuses on establishing the psychometric properties of a Portuguese version of the FOSQ-10 in a clinical population of Brazilian-Portuguese-speakers patients diagnosed with Obstructive Sleep Apnea (OSA).

## Methods

This research was conducted at the Sleep Division of the Department of Otorhinolaryngology, Faculty of Medical Sciences, State University of Campinas (Unicamp) in São Paulo, Brazil, from February 2022 to March 2023, with approval from the Institutional Research Ethics Committee (protocol CAAE: 35036620.4.0000.5404) and adhered to both International and National Guidelines. Professor Terri E. Weaver, Ph.D., RN, from the University of Illinois Chicago, 845 South Damen Avenue, MC 802, Chicago, IL 60612, granted permission to use and translate the scale. Contact e-mail: teweaver@uic.edu.

### Translation and cross-cultural adaptation

It was a prospective study, where participants diagnosed with OSA through Polysomnography (PSG), recruited from the Sleep Division of the Department of Otorhinolaryngology, including 17 women (57%) and 13 men (43%), with a mean age of 65 ± 11 years, fulfilled the FOSQ-10.

The process adhered to the guidelines proposed by Guillemin.^9^ We made two modifications to the questionnaire based on the English version: the unit of distance was changed from miles to kilometers in Questions 3 and 4, and the phrase “nod off” was culturally adapted to “pescar” (meaning “to doze” in Portuguese). The structures were evaluated, achieving a satisfactory agreement percentage (≥80%).^10^

The second part was a prospective study evaluating the reliability and validity of the Functional Outcomes of Sleep Questionnaire – Portuguese ‒ Short Version (FOSQ-10P) among Brazilian patients with OSA.

### Sample size calculation

• Cronbach's consistency,^11^ suggests indices between 0.7 and 0.9 (70%–90%). Using alpha = 0.05, Power = 90%, *k* = 10, H0 = 0.5, and Ha = 0.7, a sample size of n ≥ 92 was recommended.
• Correlation Coefficient (CO) according to G*Power 3.11,^12^ alpha = 0.05; Power = 95%; r0 = 0 (testing if r0 is significant); r1 = 0.3, suggesting a sample size of n = 112.
• Multiple Linear Regression according to G*Power 3.11^12^ with effect size f ≥ 0.15; alpha = 0.05; Power = 95%, suggesting a sample size of n = 129.
• ANOVVA according to G*Power 3.11 with an effect size f ≥ 1; alpha = 0.05; Power = 95%, suggesting a sample size of n = 22 per group.

The larger sample size, exceeding n ≥ 182, was chosen to ensure it accommodates all the analyses.

### Validation

The enrolled patients (n = 205) were referred from a Sleep Laboratory (n = 82), a sleep dentistry clinic (n = 72), and a CPAP distributor (n = 51) to the researchers. All participants suspected of OSA underwent Type I or Type II Polysomnography (PSG) based on ICSD-3/2018 Diagnostic Criteria.^13^ The PSG recording included channels of electroencephalogram, electrooculogram, chin electromyography, airflow sensors, snore microphone, respiratory effort monitoring, pulse oximetry, electrocardiogram, surface electromyography, and body position sensor. Sleep stages and respiratory events (Apnea-Hypopnea Index ‒ AHI) were scored according to the American Academy of Sleep Medicine (AASM).^14^

### Inclusion criteria

Patients over 18 years suspected of OSA^14^ and referred for polysomnography were invited to complete both scales, FOSQ-10P and ESS.

### Exclusion criteria

Participants with severe comorbidities or cognitive impairment from any cause, incomplete questionnaires (n = 4), chronic sleep deprivation, night shift workers (n = 3), and Type III cardiorespiratory monitoring (n = 5), previous treatment with Continuous Positive Airway Pressure (CPAP) and Mandibular Advancement Appliances (MAA) (n = 2) were excluded.

### Statistical analysis

We started imputing the Missing Values (MV) using the MICE package^15^ for Multivariate Imputation (MI) and employing multinomial logistic regression, suitable for our ordinal categorical data with four categories.^16^ We highlight that a response of “0” (zero value) ‒ MV ‒ in FOSQ-10P doesn't indicate greater functional impairment; instead, it means that the individual doesn’t endorse specific statements in Questions 3, 4, and 10. Therefore, caution is necessary when interpreting a response of 0.

We did an Exploratory Factor Analysis (EFA) using the Unrestricted Factor Analysis program,^17^ the Psych R package,^18^, ^19^ and The Jamovi project (Computer Software)^20^ for ANOVA, Linear Regression, and Spearman's Correlation.

The EFA aimed to assess the factorial structure of the FOSQ-10P, utilizing a polychoric matrix and the Robust Diagonally Weighted Least Squares (RDWLS).^21^ extracting the number of factors to retain relied on Eigenvalue >1,^22^ aligning with other validation studies in different languages. The Parallel Analysis (PA) technique^23^ with randomly permuted observed data was employed, and the rotation used was Robust Promin.^24^

Model fit was evaluated using the Root Mean Square Error of Approximation (RMSEA), Comparative Fit Index (CFI), and Tucker-Lewis Index (TLI) as fit indices. According to literature guidelines,^25^ RMSEA values should be below 0.08, and CFI and TLI values should be above 0.90‒0.95.

For Factor stability, we used the H index (0–1).^26^ Values (>0.80) suggest a well-defined latent variable.

A Unidimensional Congruence (UniCo) exceeding 0.95, Explained Common Variance (ECV) surpassing 0.85, and a Mean of Item Residual Absolute Loadings (MIREAL) value below 0.300 indicates that the data can be considered essentially unidimensional.^26^

### Statistics

We employed the model advocated by the American Educational Research Association^27^ ‒ “APA & NCME, 2014”, for all validation processes:

Internal Consistency (Reliability):

*Content validity:* EFA and Confirmatory Factor Analysis (CFA) of the FOSQ-10P, reporting quality indices ‒ Bartlett's test (H0 < 0.05) and Kaiser–Meyer–Olkin test (KMO ‒ H0 > 0.5); Adherence measures: Tucker & Lewis Fit Index (TLI > 0.9), Comparative Fit Index (CFI > 0.9), and Root Mean Square Error of Approximation (RMSEA ‒ H0 < 0.05);

*Construct validity:* Standardized Cronbach's Alpha (0.7‒0.9),^28^ McDonald's Omega ordinal index (0.7‒0.9),^29^ and Composite Reliability (>0.7).^30^

Validity based on relationships with external parameters:

*Convergent validity:* Spearman's correlation was computed to validate the FOSQ-10P against ESS scores ‒ <10, excessive daytime sleepiness ≥10 ESS scores (*r* ≥ 0.3).

Criterion validity: The extent to which FOSQ-10P differentiates between different AHI groups was tested (control: AHI < 5; mild: 5 ≤ AHI < 15; moderate: 15 ≤ AHI < 30; severe: AHI ≥ 30).

The sample was analyzed using ANOVA procedures to assess means across 2 or more groups. Assumptions were verified through Levene's test (*p* >  0.05), Shapiro-Wilk test (*p* > 0.05), and post hoc Tukey analysis. A *p*-value < 0.05 was considered statistically significant for the analysis, and descriptive statistics were applied to all the data.

## Results

The Translation and Cross-Cultural Adaptation of the FOSQ-10 into Brazilian Portuguese were successfully conducted, maintaining an equivalent meaning to the original English version, suitable for individuals ≥18 years old and who completed elementary education.

The population descriptive analysis of the validation process is presented in Table 1.

**Table 1:** Demographic characteristics of respondents (n = 182).

	Gender	Age	BMI	FOSQ-10P	ESS
N	Female	65	65	65	65
	Male	117	117	117	117
Mean	Female	48.3 (±12.4)	29.4 (±7.83)	14.2 (±3.65)	9.88 (±4.62)
	Male	46.9 (±12.4)	29.2 (±4.52)	15.7 (±3.25)	10.6 (±4.97)

### Factor analysis

#### Content validity

The Bartlett's sphericity test (χ² = 1108.2; df = 45; *p* =  0.000010), KMO (0.83), TLI (0.97), and CFI (0.98) indicators, as well as RMSEA (0.04), are suitable and within the expected values.

### Factor extraction ‒ Eigenvalue technique

Based on the Eigenvalue > 1 criterion, two factors were extracted with values of 5.30850 and 1.33073, while the remaining factors had Eigenvalues < 1. Both factors explain variances of 53% and 13.3%, respectively.

The items exhibited appropriate factor loadings on their respective factors. No cross-loading pattern was observed (i.e., items with factor loadings above 0.30 on more than one factor) (Table 2).

**Table 2:** Factor loadings for each item in the FOSQ10-P.

Itens	Factor 1	Factor 2
Item 1	**0.792**	−0.285
Item 2	**0.782**	−0.243
Item 3	0.682	**0.615**
Item 4	0.788	**0.508**
Item 5	**0.757**	−0.347
Item 6	**0.724**	−0.143
Item 7	**0.631**	−0.097
Item 8	**0.741**	−0319
Item 9	**0.606**	−0.254
Item 10	**0.648**	−0.232
Composite reliability	**0.891**	0.480*
H-latent	0.927	0.939
H-observed	0.900	0.874

The entries in bold are the factor loadings of each question by the Eigenvalue technique.

Indices estimate the replicability of the factor scores using the H-index suggesting that both factors could be replicable in future studies (H > 0.80).

The PA considers that only one factor extracted from the actual data will have a higher explained variance (59.4653) than random data (256.196) with indicators such as UniCo = 0.921 (0.873‒0.959), ECV = 0.822 (0.785‒0.871), and MIREAL = 0.253 (0.211‒0.282).

The missing values are non-random (linked to individuals' not performing tasks, i.e., Questions 3, 4, and 10).

In Question 3, 24.62%, and Question 4, 27.69% of female subjects responded “0” (do not perform this task), while only 1.71% of male subjects did so.

Concerning Question 10, an EFA suggested a link between MV and gender and age. A binary logistic regression confirmed the significance of both variables. We found that with each additional year of life, there is a 9% chance of not marking Question 10 compared to the previous year, and females have an 8.35% higher chance of marking “0” than males.

### Construct validity

Cronbach's alpha 0.87 (0.84‒0.9) (Table 3), and McDonald's ordinal Omega index 0.9, are satisfactory and Composite Reliability* 0.891 is suitable for a unidimensional structure.

**Table 3:** Results of internal consistency measured by Cronbach’s alpha coefficients.

Question	Mean	SD	Cronbach alpha if item deleted
1. Concentration	2.7	1.04	0.85
2. Remembering	2.9	0.95	0.86
3. Driving short distances	2.7	0.92	0.87
4. Driving long distances	2.9	1.12	0.86
5. Visiting their home	3.4	0.94	0.86
6. Relationships affected	3.5	0.87	0.86
7. Watching movies	2.6	0.98	0.86
8. Activity in the evening	2.8	1.01	0.86
9. Activity in the morning	3.0	1.09	0.87
10. Desire for intimacy	3.2	1.01	0.86
FOSQ-10P total score	15.16	3.46	0.87

### Convergent validity

Categorizing respondents assessed the FOSQ-10P according to their ESS scores were negatively correlated, (*r* = 0.364 [−0.487; −0.226]). Table 4 shows the means in the five subscales of the FOSQ-10P and the total scores for those who scored low (0 < 10) and high (≥10) on the ESS scale.

**Table 4:** Convergent validity according to ESS groups.

	ESS Groups				*p*-value	Cohen D
	ESS < 10		ESS ≥ 10			
	(n = 97)		(n = 85)			
	Mean	SD	Mean	SD		
General productivity	3.09	0.85	2.58	0.89	<0.001	0.58
Activity Level	3.31	0.71	2.91	0.76	<0.001	0.53
Vigilance	3.11	0.73	2.41	0.75	<0.001	0.93
Social Outcomes	3.53	0.81	3.21	0.99	0.019	0.35
Intimacy	3.39	0.86	2.97	1.06	0.007	0.43
FOSQ-10P	16.42	3.14	14.06	3.37	<0.001	0.71

### Criterion validity

The Spearman correlation between the AHI and FOSQ-10P was not significant (*r* = 0.026 [−0.120; 0.170]).

The ANOVA to determine whether the FOSQ-10P could discriminate subjects based on the severity of AHI was not significant (*p* = 0.144) (Table 5).

**Table 5:** Analysis of variance between the different OSA severity groups.

AIH parameters	N	Mean	SD	95% CI
Normal AHI < 5	24	13.94	4.23	(12.556; 15.329)
Mild ≤ 5 AHI < 15	48	15.298	3.403	(14.317; 16.278)
Moderate ≤ 15AHI < 30	51	15.867	3.109	(14.916; 16.818)
Severe AHI ≥ 30	59	14.939	3.392	(14.054; 15.823)

The Regression including variables from Polysomnography (AHI, Arousal Index [ArI], Wake After Sleep Onset [WASO], Oxygen Desaturation Index [ODI], Time under 90% O_2_ [T90], Body Mass Index [BMI], age, and gender) as predictors (Table 6).

**Table 6:** Regression FOSQ-10P versus PSG parameters.

PSG parameters	Estimate	SD	*t*- value	p-value
Intercept	4.87	4.39	1.11	0.269
AHI	−0.0136	0.0202	−0.67	0.504
ArI	0.0233	0.0214	1.09	0.277
Waso	−0.00222	0.00553	−0.4	0.689
ODI	0.066	0.0408	1.62	0.107
T90	−0.0414	0.0236	−1.76	0.081
BMI	0.0364	0.0511	0.71	0.478
age	0.0608	0.0206	2.95	**0.004**
Gender (male)	1.713	0.552	3.1	**0.002**

## Discussion

Our findings indicate that three out of the four selected parameters for validating FOSQ-10P ‒ content validity, construct validity, and convergent validity ‒ were thoroughly addressed.

An EFA is a specific type of Structural Equation Model (SEM) with latent variables that identify the pattern of item correlations, and the factorial structure is derived from the response to those items. Scores obtained using scales should demonstrate various indicators of validity and precision indicators^30^ that ensure their utility across different samples, populations, and over time.^31^

A widely accepted model for validity criteria, AERA, APA, & NCME, 2014,^27^ defines validity as arising from five sources of evidence: (a) Based on test content; (b) Based on response processes; (c) Based on internal structure; (d) Based on relationships with other variables (convergent, discriminant, criterion, and generalization validity); and (e) Based on consequences of testing. Therefore, our validations aimed to align with the evidence suitable for FOSQ-10P.

Validity based on Internal Consistency or reliability is a form of evidence that pertains to the empirical and theoretical structure of the instrument, revealing the latent variable through the relationships among observed variables, aiming to investigate the accuracy of the response pattern to item.^32^

Content Validity assesses how accurately a test measures the construct of interest. We found quality indices such as Bartlett's sphericity test and the Kaiser–Meyer–Olkin (KMO) and data fit indices like TLI, CFI, and RMSEA,^25^ appropriated for the use and interpretability of the scale for Brazilian-Portuguese speakers.

The Eigenvalue technique revealed two factors, consistent with a previous study.^33^ However, the emergence of the second factor was primarily due to a significant prevalence of MV - response “0” ‒ I do not perform this task ‒ in Questions 3 and 4 (short and long-distance driving), especially among female participants. Therefore, as seen in the Peruvian validation,^33^ we cannot associate this factor with Sleep-Related Impairment (SRI). But, for Question 10, analyzing MV, suggests that as years go by, both sexual desire and intimacy are affected, more among women. This may be attributed to the emphasis older men place on sexual potency, while older women see a decrease in sexual activity as a natural aspect of aging, and elderly individuals might feel uneasy discussing sexuality.^34^

The PA, where the decision on dimensionality is based on the percentage of explained common variance. Pearson's correlation was computed for linear factor analysis, and polychoric matrix was used for nonlinear factor analyses, along with the correlation of matrices. Considered the most recommended method,^35^, ^36^, ^37^ supported by its performance in comparative simulation^38^ studies and empirical applications.^23^

Thus, PA supported the single-factor structure with appropriate factor loadings, indicating that the FOSQ-10P is a unidimensional measure, as intended and confirmed by the Composite Reliability index (0.891) and the factor stability assessed through the H index (0.92).^26^ The H index assesses how well a set of items represents a common factor. Higher H values (>0.80) suggest a well-defined latent variable, likely to remain stable across different studies. Additionally, indicators such as UniCo = 0.921, ECV = 0.822, and MIREAL = 0.253 ‒ supported the unidimensionality of the scale. This aligns with Weaver's (2009)^8^ recommendation when developing the shortened version of the scale, stating that “only the total score would be used in interpreting the degree of impairment associated with daytime sleepiness”.

Construct validity is based on estimation methods that consider the importance of items through factor loadings,^25^, ^30^ indicated psychometric properties of the FOSQ-10P, such as Cronbach's α of 0.87, similar to those found in the original FOSQ-10^8^ and other validation studies.^33^, ^39^ The scale demonstrated stability even when item removal was simulated, as shown in Table 4. Additionally, indices like the McDonald's ordinal Omega index (0.9) and the Composite Reliability (0.891) support the appropriateness of interpretations and actions based on the test scores.

Validity based on the relationship to external parameters is crucial as it assesses how test scores are associated with external measures in a theoretically expected manner, examining how the construct relates to other constructs.^40^

Convergent validity demonstrates the two extents to which instruments measure similar constructs and associate them as expected. The negative correlation between FOSQ-10P and ESS, considering the inverse scoring of the scales, fell within the expected range (*r* = 0.364). The effect of FOSQ-10P results in the two groups ‒ low scores (0 < 10) and high scores (≥10) on the ESS scale, were significant (*p* < 0.05) in all five domains, as well as the total score of FOSQ-10P, with a strong effect size (Cohen's *d* 0.719), supporting convergent validity.

Criterion validity seeks validity through external criteria. For OSAS, polysomnography was the gold standard, and there was no correlation between FOSQ-10P and AHI indices. Was not possible to differentiate the groups based on the severity of AHI using FOSQ-10P. However, the impact of OSAS on quality of life may be attributed to the presence of daytime sleepiness, not just the severity of AHI, as observed in a recent study^41^ and also in ours.

We also found that younger individuals had lower FOSQ-10P scores (*p* =  0.004), suggesting that the perception of daytime sleepiness in young adults prompts them to seek investigation into sleep quality^42^ and men had a mean score of 1.616 higher on the FOSQ-10P, indicating a lower impact on daily activities than women. Possible explanations include gender differences in sensitivity to voluntary sleep deprivation^43^ and the presence of comorbid insomnia with OSA in women,^41^ along with the burden of dual responsibilities and tasks.^44^

This study's limitations are tied to administering the questionnaire exclusively before the recommended treatment, given the diverse sources of the database. The next phase of the project will involve evaluating the perception of sleepiness and functional impairment before and after different types of treatments recommended for OSA.

## Conclusion

The FOSQ-10P is a reliable and valid instrument for assessing functional status, effectively identifying significant impacts of sleep-related impairment in individuals with sleep-disordered breathing who speak Brazilian Portuguese.

## Financing

This research did not receive specific funding from public, commercial, or non-profit funding agencies.

## Conflicts of interest

The authors declare no have conflicts of interest.
